# Feasibility of robotic-assisted partial nephrectomy for complete remission of metastatic renal cell carcinoma after long exposure to immune checkpoint inhibitors (UroCCR-106)

**DOI:** 10.1093/jscr/rjac560

**Published:** 2022-12-07

**Authors:** Samy Mebroukine, Mokrane Yacoub, Clément Michiels, Alain Ravaud, Marine Gross-Goupil, Jean-Christophe Bernhard

**Affiliations:** University Hospital of Bordeaux, Urology Department, Bordeaux, France; University Hospital of Bordeaux, Pathology Department, Bordeaux, France; University Hospital of Bordeaux, Urology Department, Bordeaux, France; University Hospital of Bordeaux, Oncology Department, Bordeaux, France; University Hospital of Bordeaux, Oncology Department, Bordeaux, France; University Hospital of Bordeaux, Urology Department, Bordeaux, France

## Abstract

Immune checkpoint inhibitors used for metastatic clear cell renal cell carcinoma treatment show significant rates of complete response on metastatic sites. Feasibility of delayed surgery on primitive tumors remains questionable, especially regarding conservative procedures. We present here the first reported case of robotic-assisted partial nephrectomy (RAPN) and concomitant metastasectomy after long exposure to immunotherapy. We performed an imperative salvage RAPN and metastasectomy in a 79-year-old woman with history of right radical nephrectomy for oligometastatic clear cell renal cell carcinoma, previous open partial nephrectomy and ablative treatment on the remaining left kidney. In fact, after complete response on the metastatic sites, the patient experienced progression on the solitary kidney despite immunotherapy. This limited experience of RAPN and metastasectomy after long exposure to immunotherapy appears to be feasible safe and efficient both on the oncological and functional point of view.

## INTRODUCTION

The immune checkpoint inhibitors (ICI) era has revolutionized metastatic clear cell renal cell carcinoma (mccRCC) standard of care. ICI allows significant rate of complete radiologic response on metastatic sites [1]. However, no prospective studies have assessed delayed surgery on primitive kidney tumor. The inflammatory response due to ICI could make the surgery more complicated. The data from literature are limited, with only two retrospective studies, with small patient population size, and divergent results regarding feasibility, efficiency and safety of such procedures [2, 3]. Among the 21 cases presented through these studies, only one underwent nephron sparing surgery.

We present here the first reported case of partial nephrectomy (PN) and metastasectomy post-ICI with evaluation of feasibility, surgical outcomes and efficiency of such procedure.

## CASE REPORT

The clinical data was collected, after patient’s consent, from the prospective database UroCCR (CNIL DR 2013-206; NCT03293563).

**Figure 1:**
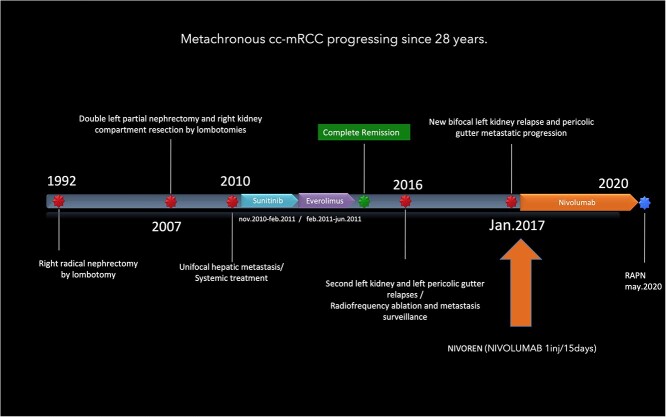
Timeline of a metachronous cc-mRCC progressing in the last 28 years.

This is the case of a 79-year-old woman, incidentally diagnosed, 30 years ago, with a 5 cm, Fuhrman grade I, clear cell renal cell carcinoma (ccRCC) and treated by right open radical nephrectomy (RN; Fig. 1). Pathological stage was pT1b N0 M0 R0.

Fifteen years after initial treatment, a biopsy confirmed multifocal relapse was diagnosed on the right nephrectomy site (4.5-cm lesion) and on the contralateral solitary kidney (4 and 1-cm tumors). Two successive open surgeries were done, including a double tumorectomy on the left kidney and a renal fossa revision. The specimen pathological analysis reported on the left kidney, two Fuhrman grade I ccRCCs and a Fuhrman grade II relapse on the right side, with negative surgical margins in both cases.

Three years later, a single liver metastasis located in the sixth segment was confirmed by biopsy, leading to a systemic treatment by SUNITINIB, interrupted after 3 months for adverse events including severe acute thrombopenia. This led to the introduction of EVEROLIMUS for 5 months. A complete radiologic response was established. Subsequently, a second posterior and equatorial relapse on the left kidney was diagnosed, along with a retroperitoneal metastasis of the left paracolic gutter. An ablative treatment was performed on the kidney tumor and the paracolic nodule was at first followed. Four months after ablative treatment, a third bifocal local recurrence on the kidney led to the inclusion of the patient in the NIVOREN study [4] with a bimonthly NIVOLUMAB 225 mg injection.

**Figure 2:**
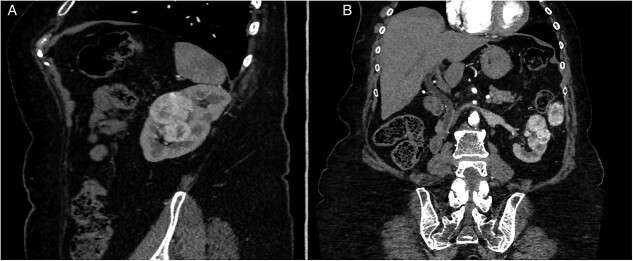
Preoperative CT-scan. (**A)** Sagittal section. (**B**) Modified coronal section allowing to see the left pericolic gutter metastasis.

After 40 months of nivolumab exposure, no distant relapse was experienced. However, the three known lesions of the kidney and left paracolic gutter progressed in size and led to the decision of an imperative robotic-assisted double partial nephrectomy (RAPN) and concomitant metastasectomy. The patient’s preoperative assessment was Eastern Cooperative Oncologic Group (ECOG) performance status grade 0, an American Society of Anesthesiologists (ASA) physical status grade 3, with a 29-body mass index (BMI) and a stage IV renal chronic disease (serum Creatinine, sC: 156 μmol/l, modification of diet for renal disease (MDRD) equation of glomerular filtration rate (GFR): 27 ml/min/1.73 m^2^). The tumor stage was cT1b cN0 cM1. The renal tumor was considered a merging of two kidney lesions, measuring in total 59 mm in diameter and totally equatorial. The paracolic gutter metastasis was 40 mm in diameter (Fig. 2). The RENAL nephrometry and PADUA scores were 10ah and 11a, respectively.

**Figure 3:**
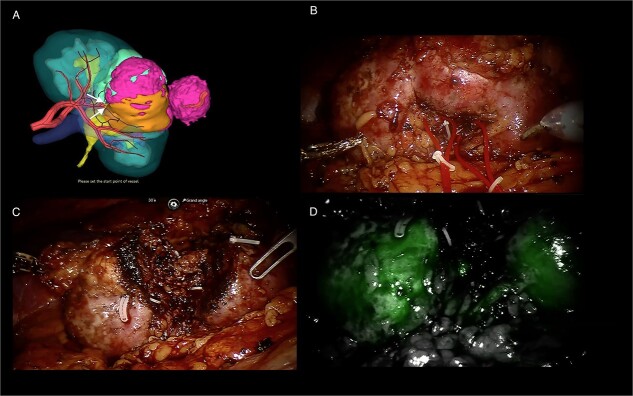
3D-model (FUJIFILM) of the tumoral kidney and per operative screenshots. (**A**) 3D-model (FUJIFILM) of the tumoral kidney with superselective clamping points. (**B**) Tumoral kidney before tumorectomy. (**C**) Kidney after tumorectomy. (**D**) Indocyanin green angiography after tumorectomy with homogeneous parenchyma coloration.

The Synapse 3D^®^ software (Fujifilm) was used to make tridimensional reconstructions of the tumoral kidney. Three-Dimensional Image Guided Surgery (3D-IGRAPN), as previously described [5], was performed with the Da Vinci Surgical Robot (Intuitive Surgical). Because of the history of partial nephrectomy, we decided not to dissect the vascular pedicle and directly target tumor-feeding arterial branches in the hilum as identified on the preoperative 3D Model (Fig. 3). Fourth order arterial branches superselective clamping and the expected induced ischemia was simulated. Operating time was 5 h, estimated blood loss was 1000 ml under superselective arterial clamping of 36 min. Technical tips of this minimally invasive salvage nephron sparing surgery with metastasectomy after long exposure to immunotherapy are reported in the following video: https://www.youtube.com/watch?v=q1lxSd2VHUc.

Regarding feasibility and considering the history of PN and radiofrequency ablation, kidney mobilization, tumor enucleation and metastasectomy did not seem to bear any additional difficulties after immunotherapy.

Postoperative functional outcomes showed a sC peak at Day 3 (215 μmol/l) and discharge sC at Day 6 was 188 μmol/l. No postoperative complication occurred in the 30 days following surgery. The specimen analysis reported a 4.8-cm Fuhrman grade III ccRCC with tumor thrombus in a segmental vein. Metastasis was a 3.5-cm nodule of Fuhrman grade III ccRCC. Both were resected with negative surgical margins. Tumor and peritumoral lymphocyte infiltration was assessed with a predominant CD4+ T-cell population with CD4+/CD8+ ratio at 3. Low PD-1 and PD-L1 (both <5%) tumor expressions were found. Final stage was pT3a cN0 pM1 R0. Systemic treatment was not reintroduced and at last follow-up of 21 months, the patient was still recurrence-free with a kidney function comparable to preoperative (sC: 160.5μmol/l; MDRD GFR: 28.5 ml/min).

## DISCUSSION

This case report emphasizes the feasibility and safety of minimally invasive nephron sparing surgery for complete remission of mRCC after long exposure to ICI, even in difficult conditions such as salvage situation on a solitary kidney. Despite a limited experience in the international literature, with divergent conclusions on surgical outcomes and safety of RN in this setting, conservative approaches may be part of the offered options for selected cases and in expert centers [2, 3]. Moreover, on the oncologic side, negative surgical margins were achieved, and this strategy led to systemic treatment interruption and mid-term complete remission. Although, some experiences of pT0 on the kidney were reported [2], this case conversely stressed out a dissociated response to ICI between metastatic sites and the kidney tumor. This questions on the interest of predictive biomarkers such as PD-L1 tumor status to monitor the use of anti-PD-1 immunotherapy. Several studies focusing on neoadjuvant immunotherapy [6, 7] or evaluating ICI’s impact on primary tumors in mRCC [8, 9] have shown limited effect. These data are in accordance with the present case where the primary tumor was progressing despite a well-controlled disease at distant sites. Finally, this case report also highlights how complementary multiple lines of systemic treatments and local focal procedures are in long-term oligometastatic RCC management. This strategy resulted in a 30 years prolonged disease control and further off-treatment and tumor free survival.

## CONCLUSION

With expertise, RAPN after ICI appears to be feasible safe and efficient both on the oncological and functional point of view. It could be an interesting option for selected cases of mRCC with indication of delayed nephrectomy to obtain complete remission.

## CONFLICT OF INTEREST STATEMENT

The authors have no conflict of interests to declare.

## FUNDING

This article was supported by the “Fonds pour la recherche et l’innovation en chirurgie rénale - Fondation Bordeaux Université”.

## DATA AVAILABILITY

Data are available in the UroCCR Database (CNIL DR 2013-206; NCT03293563) as mentioned in the manuscript.
